# The Life Story Practice, Education, and Research Collaborative: Program Needs and Development

**DOI:** 10.1093/geroni/igaf122.3407

**Published:** 2025-12-31

**Authors:** Millicent Malcolm, Juliette Shellman

**Affiliations:** University of Connecticut, Storrs, Connecticut, United States; University of Connecticut, Farmington, Connecticut, United States

## Abstract

Life stories play a vital role in promoting the health and well-being of older individuals, families, and communities. Formal training is essential for delivery of safe and effective life story work. The Life Story Collaborative, an emerging program of The International Center for Life Story and Innovations, aims to foster the growth of knowledge, skills, and interdisciplinary partnerships in Life Story education, research, and practice. A needs assessment conducted at our inaugural meeting identified learning needs, interests, participation goals, program suggestions, and meeting time preferences. Participants (N = 15) were from across the United States, the Netherlands, and Japan representing 5 time zones. Ten of fifteen participants completed the survey. The group consisted of academic researchers and educators, clinicians, ethnographers, and product and service innovators. Participants shared interests for professional development in life story approaches to enhance health outcomes in older adults including end-of-life, safe practices with culturally diverse and underrepresented groups, networking and resource sharing, and disseminating work to advance and draw attention to the field. Suggestions for future meetings included specific guest speakers and topics such as use of artificial intelligence, brain research, practice methods, and funding mechanisms. Positive comments were shared about the meeting and future directions for this group. Results indicate there is a wide variety of interests in the life story field, but few opportunities for formal learning, engagement and collaboration. We have taken the first step to fill this gap with a needs assessment that will guide the development and implementation of The Life Story Collaborative.

